# Potential of Marine Bacterial Metalloprotease A69 in the Preparation of Antarctic Krill Peptides with Multi-Bioactivities

**DOI:** 10.3390/md23060226

**Published:** 2025-05-24

**Authors:** Rui Liu, Wen-Jie Cao, Wen-Xiao Zhao, Xiao-Jie Yuan, Yu-Zhong Zhang, Qi-Long Qin, Xiao-Yan Song, Xi-Ying Zhang, Jian Li, Xiu-Lan Chen, Yu-Qiang Zhang

**Affiliations:** 1 State Key Laboratory of Microbial Technology, Marine Biotechnology Research Center, Shandong University, Qingdao 266237, China; liurui7317@163.com (R.L.); 202232574@mail.sdu.edu.cn (W.-J.C.); 202132652@mail.sdu.edu.cn (W.-X.Z.); yuanxj0906@163.com (X.-J.Y.); zhangyz@sdu.edu.cn (Y.-Z.Z.); qinqilong@sdu.edu.cn (Q.-L.Q.); xysong@sdu.edu.cn (X.-Y.S.); zhangxiying@sdu.edu.cn (X.-Y.Z.); 2 Frontiers Science Center for Deep Ocean Multispheres and Earth System & College of Marine Life Sciences, Ocean University of China, Qingdao 266003, China; 3 Joint Research Center for Marine Microbial Science and Technology, Shandong University and Ocean University of China, Qingdao 266237, China; 4 Laboratory for Marine Biology and Biotechnology, Qingdao Marine Science and Technology Center, Qingdao 266237, China

**Keywords:** marine bacterial protease A69, Antarctic krill peptides, antioxidant activity, angiotensin-converting enzyme (ACE)-inhibitory activity, antibacterial activity, α-glucosidase-inhibitory activity, dipeptidyl peptidase IV (DPP-IV)-inhibitory activity

## Abstract

Antarctic krill (*Euphausia superba*) is a nutrient-rich marine resource. Although several terrestrial proteases have been used to prepare Antarctic krill peptides (AKPs), there has been no report on the preparation of AKPs using a marine protease. Here, marine bacterial protease A69 was used to prepare AKPs with multi-bioactivities. Through optimizing hydrolysis parameters, we established a process for AKPs preparation by hydrolyzing Antarctic krill powder with A69. In the prepared AKPs, peptides less than 3000 Da and 1000 Da accounted for 99.23% and 88.37%, respectively. The scavenging ratios of the AKPs to ABTS^+^, DPPH· and ·OH reached 93.23 ± 0.09%, 99.90 ± 0.15%, and 93.90 ± 0.47%, respectively. The AKPs also had high angiotensin-converting enzyme (ACE)-inhibitory activity, with an IC_50_ of 0.22 ± 0.04 mg/mL. At 40 mg/mL, the AKPs inhibited α-glucosidase and dipeptidyl peptidase IV (DPP-IV) activities by 7.18% and 13.62%, respectively, and displayed antibacterial activity to *Escherichia coli*. Moreover, 14 antioxidant peptides, 24 ACE-inhibitory peptides, 2 α-glucosidase-inhibitory peptides, and 10 DPP-Ⅳ-inhibitory peptides were identified from the AKPs. These results demonstrate that the prepared AKPs contain diverse bioactive peptides and have multi-bioactivities. This study indicates that marine bacterial protease A69 has promising application potential in preparing AKPs with multi-bioactivities.

## 1. Introduction

Active peptides, consisting of 2 to 50 amino acid residues, have a variety of biological activities, and have been widely used as bioactive ingredients in the fields of food [1,2], medicine [3], healthcare products [1,4] and others. Active peptides have been shown to have a variety of biological functions, such as antioxidant [5,6,7], anti-inflammatory [8,9], antibacterial [10,11,12], immune regulation [13,14,15], lowering blood pressure [16,17] and lowering uric acid [18], etc. Active peptides are now commonly prepared by enzymatic hydrolysis technology. Many proteases from terrestrial animals, plants and microorganisms have been used in the preparation of active peptides, including pepsin, trypsin, papain, alkaline protease, neutral protease and others [19,20]. In contrast, marine-derived proteases used in the preparation of active peptides are still limited.

Marine environments harbor more than 80% of global biodiversity, and serve as a vast reservoir of novel enzymes and bioactive compounds [21,22]. Marine-derived bioactive compounds have been shown to have anti-inflammatory [23], antimicrobial [24,25], anticancer [26,27] and other activities, some of which have been developed into FDA-approved drugs [25,27]. Antarctic krill (*Euphausia superba*) is a small crustacean widely distributed in Antarctic waters. As a key species in the Antarctic marine ecosystem, Antarctic krill is not only an important part of the Antarctic food chain and provides a major food source for a wide variety of marine life [28], but also an important source of high-value bioactive substances. Antarctic krill has been used as raw material for the production of astaxanthin [29,30], chitin [31,32], krill oil [33,34,35], krill peptides [34,36], and feed [35,37,38], etc. In addition, Antarctic krill has attracted more and more attention in the food [36,39,40,41], healthcare [34,42] and pharmaceutical fields [42,43,44] due to its rich nutritional composition and bioactive characteristics. Although some terrestrial proteases, including neutral protease, alkaline protease, Corolase PP, trypsin, pepsin, papain, flavor protease, thermoase PC10F, protamex and animal proteolytic enzyme, have been used in the preparation of Antarctic krill peptides (AKPs), which have antioxidant, anti-hypertensive, anti-diabetic and/or antibacterial activities [45,46,47,48,49,50,51,52,53], there has been no report on the preparation of Antarctic krill peptides using marine-derived proteases.

Protease A69 is a MEROPS M4 family metalloprotease derived from the marine bacterium *Anoxybacillus caldiproteolyticus* 1A02591 [54]. In previous studies, protease A69 was heterologously expressed in *Escherichia coli* and *Bacillus subtilis*, and recombinant protease A69 has an apparent molecular weight of approximately 34 kDa, an optimal temperature of 60 °C, and an optimal pH of 7.0 [54,55]. Recombinant protease A69 has been used to prepare bovine collagen peptides [54], soy protein peptides [55], and peanut peptides [56]. During protein hydrolysis, protease A69 preferentially hydrolyzes peptide bonds with hydrophobic residues at the P1’ site, such as Leu, Phe, Ile, Val, and Ala, and the prepared peptides exhibit bioactivities. The prepared bovine collagen peptides have moisturizing ability and antioxidant activity, the prepared soybean peptides have angiotensin-converting enzyme (ACE)-inhibitory activity, and the prepared peanut peptides have antioxidant activity and ACE-inhibitory activity. These studies showed that the marine-derived protease A69 has good potential in preparing bioactive peptides.

The aim of this study was to evaluate the potential of marine bacterial protease A69 in the preparation of AKPs with biological activities. With Antarctic krill powder, a by-product from krill oil production, as the material, on the basis of optimizing hydrolysis parameters, a process of preparing AKPs by hydrolyzing Antarctic krill powder with protease A69 was established. The characteristics and biological activities of the prepared AKPs were further analyzed. The AKPs have high proportion of low molecular weight peptides, contain 20 amino acids, and have antioxidant, ACE-inhibitory, α-glucosidase-inhibitory, dipeptidyl peptidase IV (DPP-IV)-inhibitory and antibacterial activities. The results show that the marine bacterial protease A69 has great application potential in the preparation of AKPs with good nutritional function, and antioxidant, anti-hypertensive, anti-diabetic and antibacterial activities.

## 2. Results and Discussion

### 2.1. Optimization of the Hydrolysis Parameters of Protease A69 on Antarctic Krill Powder

**Figure 1:**
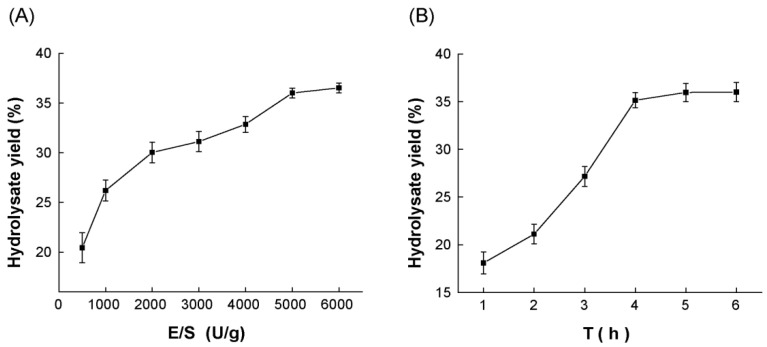
Optimization of the parameters for protease A69 to hydrolyze Antarctic krill powder. (**A**) The effect of the E/S ratio on the hydrolysate yield. Antarctic krill powder was hydrolyzed at 60 °C and pH 7.0 for 6 h by A69 under different E/S ratios. (**B**) The effect of hydrolysis time on the hydrolysate yield. Antarctic krill powder was hydrolyzed at the E/S ratio of 5000 U/g, 60 °C, and pH 7.0 for different time durations. The graphs show data from triplicate experiments (mean ± SD).

Protease A69 was previously shown to achieve the highest activity at 60 °C and pH 7.0 [55]. To determine the optimal conditions for A69 to hydrolyze Antarctic krill powder for AKPs production, two additional enzymatic hydrolytic parameters, enzyme/substrate (E/S) ratio and hydrolysis time, were optimized at 60 °C and pH 7.0 by single-factor experiments. As shown in Figure 1A, when Antarctic krill powder was hydrolyzed with A69 at the E/S ratio range from 500 U/g to 6000 U/g, the hydrolysate yield increased rapidly with the E/S ratio from 500 U/g to 5000 U/g, but only showed a slight increase from 5000 U/g to 6000 U/g. In addition, the content of oligopeptides with a molecular weight < 500 Da in the hydrolysates from 5000 U/g and 6000 U/g hydrolysis was almost the same, reaching the highest (Table 1). Based on these results, the optimal E/S ratio for A69 to hydrolyze Antarctic krill powder for AKPs production was determined to be 5000 U/g. As shown in Figure 1B, the hydrolysate yield increased with the hydrolysis time and reached the peak at 5 h. The content of oligopeptides with a molecular weight < 1000 Da in the hydrolysate from 6 h hydrolysis was the highest, and the contents of those < 500 Da were similar in the hydrolysates from 5 h and 6 h hydrolysis (Table 2). Based on these results, the optimal hydrolysis time for A69 to hydrolyze Antarctic krill powder for AKPs production was determined to be 6 h.

Protease A69 was previously used to prepare active peptides from bovine bone collagen, soy protein, and peanut protein. The optimal E/S ratio and hydrolysis time for recombinant A69 from *E. coli* to prepare collagen peptides were determined to be 25 U (collagenolytic activity)/g, and 2 h, respectively [54]; those for recombinant A69 from *B. subtilis* to prepare soy protein peptides were 4000 U (caseinolytic activity)/g, and 3 h [55]; and those for recombinant A69 from *B. subtilis* to prepare peanut protein peptides were 3000 U (caseinolytic activity)/g, and 4 h [56]. In this study, the optimal E/S ratio and hydrolysis time for recombinant A69 from *B. subtilis* to prepare AKPs from Antarctic krill powder were determined to be 5000 U (caseinolytic activity)/g, and 6 h. These differences may be attributed to the discrepancy in the sequences and structures of these protein sources.

In addition, it is worth mentioning that, in this study, the freeze-dried supernatant derived from Antarctic krill powder hydrolysis was used to determine the hydrolysate yield in hydrolysis parameter optimization. In addition to peptides, the supernatant may contain soluble non-peptide components, such as inorganic salts, free amino acids, and others from Antarctic krill powder during hydrolysis.

### 2.2. Preparation and Characterization of AKPs

**Figure 2:**
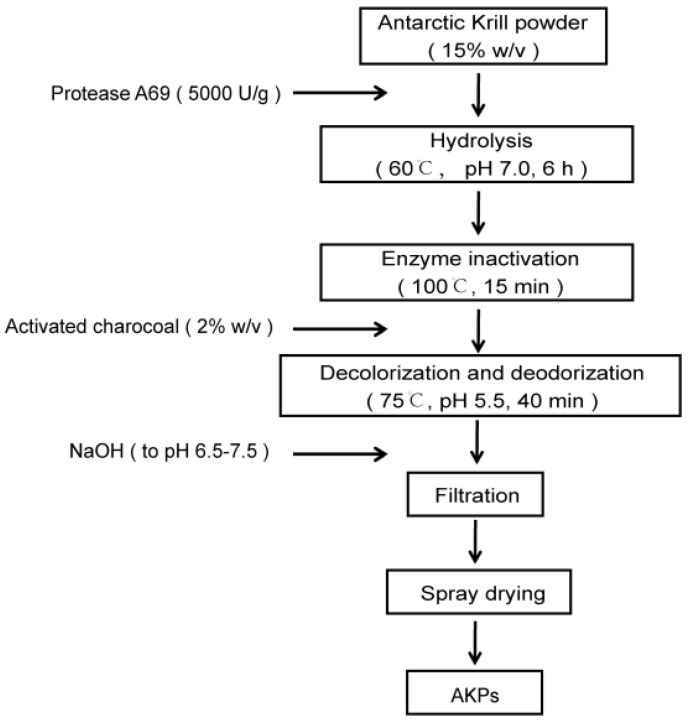
A flow chart of AKPs preparation with protease A69.

**Figure 3:**
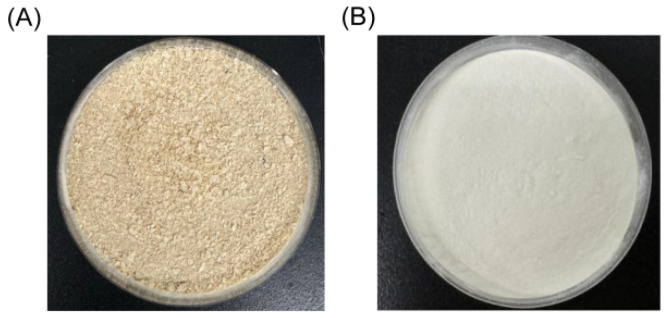
Antarctic krill powder (**A**) and the AKPs powder (**B**). The AKPs powder was prepared using the process shown in Figure 2.

**Figure 4:**
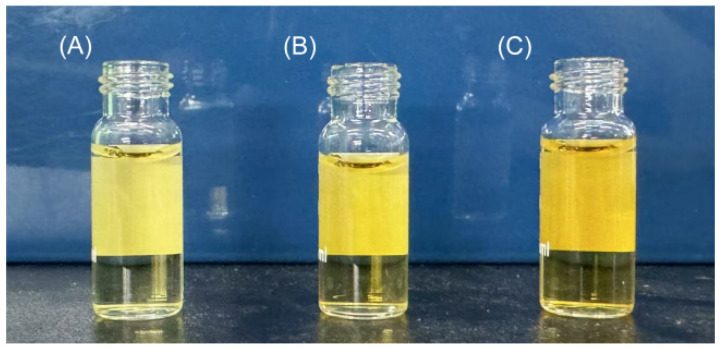
Solubility of the prepared AKPs powder. (**A**) The AKPs solution of 10% (*w*/*v*) concentration. (**B**) The AKPs solution of 20% (*w*/*v*) concentration. (**C**) The AKPs solution of 30% (*w*/*v*) concentration.

**Figure 5:**
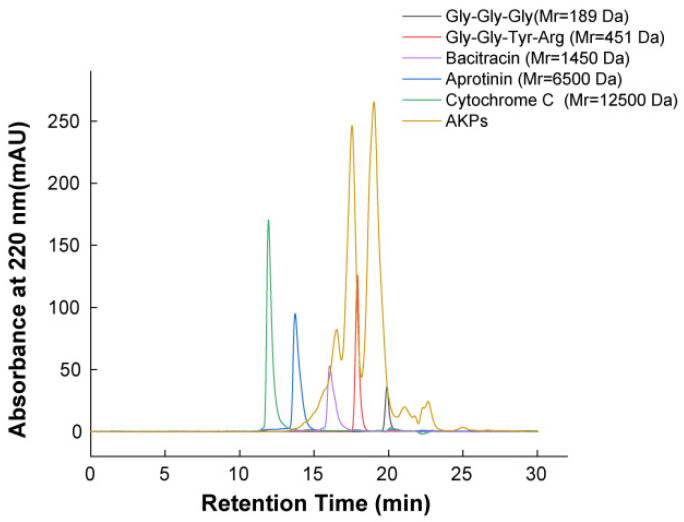
Molecular weight distribution of the prepared AKPs analyzed by HPLC gel filtration.

Based on the optimal hydrolysis parameters determined above, we set up a process to prepare AKPs using protease A69, and a flow chart of this process was shown in Figure 2. The AKPs prepared with this process were milky white powder (Figure 3) and had good water solubility when the concentration reached 30% (*w*/*v*) (Figure 4). The enzymatic hydrolysis efficiency of the Antarctic krill powder was 35.99 ± 0.03% based on its weight before and after enzymatic hydrolysis, and 60.83 ± 0.02% based on its protein content before and after enzymatic hydrolysis. The molecular weight distribution of peptides in the AKPs prepared with the established process were further analyzed. The result showed that peptides with a molecular weight < 500 Da in the AKPs accounted for 52.37% and the content of those < 1000 Da was 88.37% (Figure 5 and Table 3), indicating that the prepared AKPs were rich in peptides composed of less than 10 amino acid residues. It has been reported that bioactive peptides usually contain 2–20 amino acid residues [1]. Thus, the prepared AKPs may contain a variety of bioactive peptides with various biological activities.

### 2.3. Amino Acid Composition of the Prepared AKPs

To evaluate the nutritional value of the prepared AKPs, the free and total amino acid composition of the AKPs was detected. The content of free amino acids in the prepared AKPs was less than 5%, and that of total amino acids was more than 63% (Table 4). A total of 17 amino acids were detected after acid hydrolysis. However, because Asn and Gln were, respectively, converted to Asp and Glu, and Trp was degraded during acid hydrolysis, the actual total amino acid composition of the AKPs was likely comprised of 20 amino acids. Among the total amino acids in the AKPs, the detected contents of acidic amino acids Glu and Asp were quite high, accounting for 9.80 ± 0.72% and 6.58 ± 0.54%, which, however, should be the total contents of Glu + Gln and Asp + Asn, respectively. The contents of Pro and Lys were also high, reaching 8.11 ± 0.50% and 5.31 ± 0.49%, respectively. In addition, eight human essential amino acids were identified in the AKPs, totally accounting for 22.74 ± 0.27%. Considering that Trp was destroyed during acid hydrolysis, the AKPs actually contain all nine human essential amino acids. These results show that the prepared AKPs have high nutritional value.

### 2.4. The Antioxidant Activity of the Prepared AKPs

**Figure 6:**
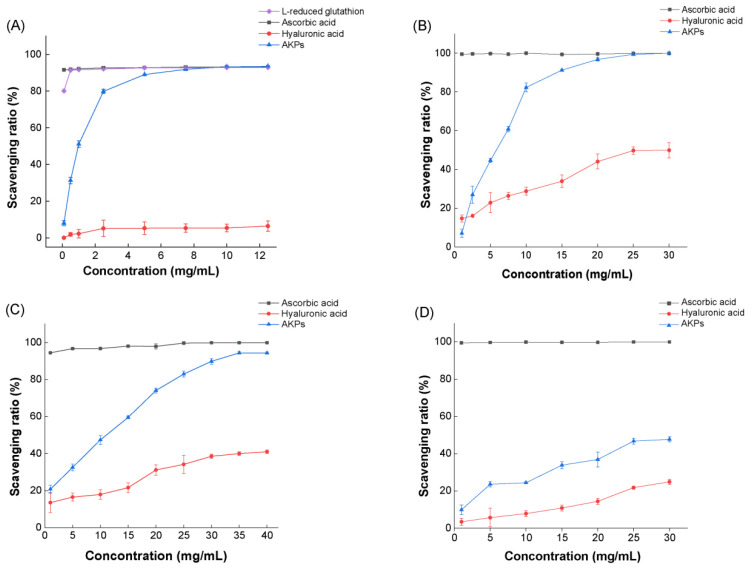
Antioxidant activity of the prepared AKPs. (**A**) ABTS^+^ scavenging capacity of the AKPs. (**B**) •OH scavenging capacity of the AKPs. (**C**) DPPH• scavenging capacity of the AKPs. (**D**) O_2_^−^• scavenging capacity of the AKPs. The graphs show data from triplicate experiments (mean ± SD).

The in vitro antioxidant activity of the prepared AKPs was evaluated by determining its scavenging ratios to free radicals ABTS^+^, DPPH·, ·OH and O_2_^−^·. Ascorbic acid (Vc), hyaluronic acid (HA), and L-reducing glutathione were used as three positive controls in determining the ABTS^+^ scavenging ratio, while Vc and HA were used as two positive controls in determining the scavenging ratios of DPPH·, ·OH and O_2_^−^·. As shown in Figure 6, within the investigated range of the AKPs concentration, the scavenging ratios of all free radicals rose with the increase in the AKPs concentration, demonstrating a dose-effect scavenging ability of the AKPs to the radicals. The scavenging ratio to ABTS^+^ of the AKPs at 10 mg/mL reached 93.23 ± 0.09%, approximately the same as those of Vc and L-reducing glutathione, and much higher than that of HA (Figure 6A). The scavenging ratios of the AKPs to DPPH· and ·OH were also high, reaching 99.90 ± 0.15% (at 30 mg/mL), and 93.90 ± 0.47% (at 40 mg/mL), respectively, basically equivalent to those of Vc, and much higher than those of HA (Figure 6B,C). By contrast, the scavenging ratio of the AKPs to O_2_^−^·was much lower, reaching 47.70 ± 1.38% at 30.00 mg/mL (Figure 6D). The EC_50_ values of the AKPs were 0.93 ± 0.02 mg/mL for ABTS^+^, 9.04 ± 0.63 mg/mL for DPPH· and 5.10 ± 0.18 mg/mL for ·OH. Altogether, these results indicated that the AKPs possess strong scavenging ability to a variety of free radicals, showing good antioxidant activity.

Several studies have shown that Antarctic krill hydrolysates (AKHs) prepared using terrestrial enzymes have antioxidant activity. Zhang et al. reported that the ·OH and DPPH· scavenging ratios of the AKH prepared using alcalase were 65.99 ± 1.22% and 55.32 ± 1.08% at 5 mg/mL, respectively, significantly higher than those prepared using trypsin, Neutrase, pepsin or papain [51]. Lan et al. reported that the high Fischer ratio oligopeptides, prepared from the sequential hydrolysis of Antarctic krill powder with alcalase and flavorzyme, showed scavenging activity against four free radicals, with EC_50_ values of 0.91 mg/mL for ABTS^+^, 0.83 mg/mL for O2-·, 4.90 mg/mL for DPPH·, and 4.62 mg/mL for ·OH [47]. Our results in this study showed that the AKPs prepared using marine bacterial protease A69 had a comparable EC_50_ value for ABTS^+^ (0.93 ± 0.02 mg/mL), and higher EC_50_ values for DPPH· (9.04 ± 0.63 mg/mL) and ·OH (5.10 ± 0.18 mg/mL).

### 2.5. The ACE-Inhibitory Activity of the Prepared AKPs

**Figure 7:**
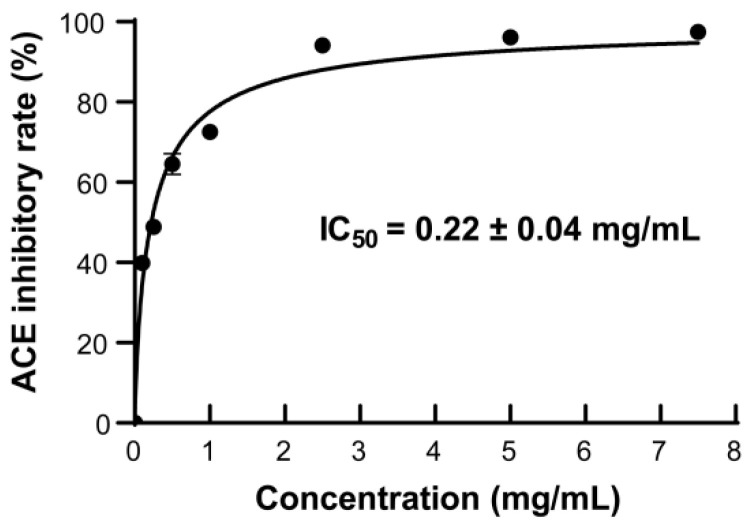
The ACE-inhibitory activity of the AKPs. The graph shows data from triplicate experiments (mean ± SD).

ACE inhibitors have been used to treat hypertension by blocking the activity of ACE in the renin–angiotensin–aldosterone system of humans [57]. Many peptides with ACE-inhibitory activity have been reported, such as tuna muscle peptide [16], broccoli peptide [58], walnut peptide [59], etc. In order to ascertain the ACE-inhibitory activity of the AKPs prepared with protease A69, the ACE-inhibitory rates of the AKPs of different concentrations were measured. As illustrated in Figure 7, the ACE-inhibitory rate increased promptly with the AKPs concentration and reached 94.12 ± 0.45% at 2.5 mg/mL. The IC_50_ value of the AKPs for ACE was determined to be 0.22 ± 0.04 mg/mL. Thus, the AKPs demonstrated remarkable ACE-inhibitory activity and likely contain ACE-inhibitory peptides.

It has been reported that AKHs prepared with commercial enzymes have ACE-inhibitory activity. The ACE-inhibitory rate of the AKH prepared with trypsin was 38.82 ± 0.71% when the concentration was 1 mg/mL [45]. The IC_50_ of the AKH prepared by hydrolysis of peeled Antarctic krill tail meat using thermoase PC10F was 1.944 mg/mL [48]. Ji et al. reported that the ACE-inhibitory rate of the AKH prepared with corolase PP was approximately 48% at 10 mg/mL, which was higher than those prepared with alcalase, flavourzyme, and papain [53]. The IC_50_ value (0.22 ± 0.04 mg/mL) of the AKPs prepared with marine bacterial protease A69 in this study is much lower than those of the reported AKHs prepared with terrestrial commercial enzymes, suggesting that the AKPs have higher ACE-inhibitory activity.

### 2.6. The Antibacterial Activity of the Prepared AKPs

**Figure 8:**
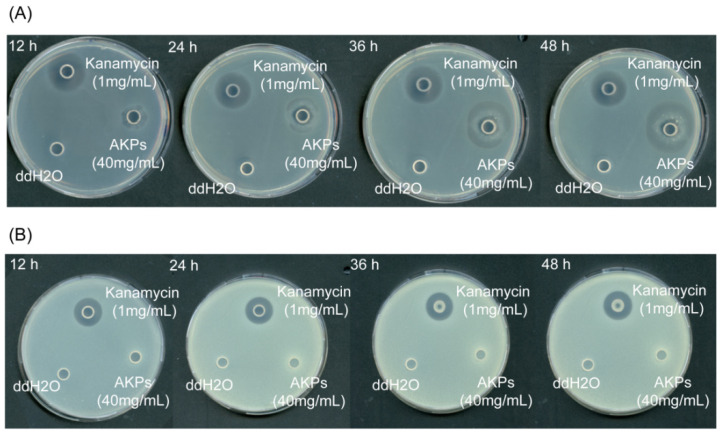
Antibacterial effects of the AKPs on *E. coli* (**A**) and *S. aureus* (**B**). Plates in the figure are representatives from triplicate experiments.

To detect the antibacterial activity of the AKPs, the antibacterial effects of the AKPs on *E. coli* and *S. aureus* were studied by observing the inhibition zone formation on solid agar plates. As shown in Figure 8, the AKPs at 40 mg/mL formed a clear inhibition zone on the plate containing *E. coli* cells after 12 h incubation, and the diameter of the zone increased with incubation time, reaching 2.99 ± 0.08 mm at 48 h, in contrast to that of the positive control kanamycin (1 mg/mL) that retained approximately the same size (2.38 ± 0.20 mm) from 12 to 48 h (Figure 8, Table 5). On the plate containing *S. aureus* cells, the positive control kanamycin (1 mg/mL) formed a clear inhibition zone, but the AKPs did not (Figure 8). This result showed that the AKPs had antibacterial activity against the Gram-negative bacterium *E. coli*, but not against the Gram-positive bacterium *S. aureus*.

Zhao et al. reported that the AKH prepared with protamex showed antibacterial activity against *S. aureus* [49]. In contrast, our study showed that the AKPs prepared with protease A69 exhibited antibacterial activity against *E. coli*, but no antibacterial activity against *S. aureus*. This difference suggests that proteases protamex and A69 likely have distinct hydrolytic sites on Antarctic krill proteins and generate bioactive peptides with differences in amino acid composition and sequence, structural characteristics and antimicrobial activity.

### 2.7. The Inhibitory Activities of the AKPs to α-Amylase, α-Glucosidase and DPP-IV

To evaluate the hypoglycemic potential of the AKPs, its inhibitory effects on α-amylase, α-glucosidase and DPP-Ⅳ activities were measured. When the concentration of AKPs was 40 mg/mL, its inhibitory rates on α-glucosidase and DPP-Ⅳ activities were 7.18% and 13.62%, respectively, and it had no inhibitory effect on α-amylase activity.

There has been no report on the α-amylase-inhibitory activity of AKH, and only one report on the α-glucosidase-inhibitory activity of AKH. Zheng et al. reported that the α-glucosidase-inhibitory ratio of the AKH prepared with neutral protease was 43.82%, which was higher than that of AKH prepared with trypsin, alcalase, protamex, papain or flavoenzyme [46]. In contrast, there are more reports on the DPP-Ⅳ-inhibitory activity of AKHs prepared with different enzymes. Ji et al. reported that the AKH prepared with an animal proteolytic enzyme showed DPP-Ⅳ-inhibitory activity with an IC_50_ value of 1.6272 mg/mL [50]. They also prepared AKHs using corolase PP, alcalase, flavourzyme, and papain, and found that the AKH prepared with corolase PP had the highest inhibition rate against DPP-IV, approximately 40% at a concentration of 10 mg/mL [53]. Lang et al. reported that the AKHs prepared with compound protease, neutral protease, alkaline protease, flavor protease, and animal hydrolase all had inhibitory activity against DPP-IV at a concentration of 100 mg/mL, and that preparation with compound protease displayed the highest value (66.81 ± 2.50%) [52]. Similar to these reports, our results in this study showed that the AKPs prepared with marine bacterial protease A69 had inhibition effects on α-glucosidase and DPP-IV activities, but not on α-amylase activity.

### 2.8. Identification of Bioactive Peptides from the Prepared AKPs

The peptide sequences in the prepared AKPs were determined by LC-MS/MS, and a total of 5657 peptide sequences from the AKPs were detected. By searching the AODB database, fourteen antioxidant peptides were identified from the detected peptide sequences in the prepared AKPs, including three dipeptides, five tripeptides, two tetrapeptides, one pentapeptide, one hexapeptide, and two heptapeptides (Table 6). Similarly, twenty-four ACE-inhibitory peptides were identified by searching the AHTPDB database, including eight dipeptides, eight tripeptides, six tetrapeptides, and two pentapeptides (Table 7). By searching the BIOPEP-UWM database, two α-glucosidase-inhibitory peptides (one pentapeptide and one heptapeptide) (Table 8) and ten DPP-Ⅳ-inhibitory peptides (seven dipeptides and three tetrapeptides) (Table 9), but no α-amylase-inhibitory peptide, were identified. In addition, no antimicrobial peptide was identified by searching the APD database. These data demonstrated that the AKPs prepared with protease A69 contain diverse bioactive peptides, consistent with its multiple bioactivities determined above.

In the AODB database, no antioxidant peptides of Antarctic krill origin were deposited. The 14 antioxidant peptides we identified from the prepared AKPs show homolog sequences to those previously reported from other animals and plants, marking their first identification from Antarctic krill. In the AHTPDB database, there are four ACE-inhibitory peptides from Antarctic krill currently documented, including VW [48,85], LKY [48,85], ITRY [85], and VFER [85]. In this study, we identified 24 ACE-inhibitory peptides from the prepared AKPs that differ from known Antarctic krill-derived sequences in the database, representing their first discovery in Antarctic krill. In the APD database, no antimicrobial peptides from Antarctic krill were recorded, and none were identified from the prepared AKPs through searching this database. However, the prepared AKPs exhibited in vitro antibacterial activity against *E. coli* (Figure 8), suggesting that there are antimicrobial peptides in the AKPs that could not be identified through database search, which thus need future identification. By searching the BIOPEP-UWM database, no α-amylase-inhibitory peptides were identified from the prepared AKPs, consistent with the lack of in vitro α-amylase-inhibitory activity of the AKPs. However, α-glucosidase-inhibitory peptides and DPP-Ⅳ-inhibitory peptides were identified from the AKPs, consistent with the inhibitory effects of the AKPs on α-glucosidase DPP-Ⅳ activities detected above. Notably, the inhibitory effect of the AKPs on α-glucosidase activity was quite low, probably due to the small number and/or low abundance of α-glucosidase-inhibitory peptides in the AKPs.

## 3. Materials and Methods

### 3.1. Experimental Materials

Antarctic krill powder was kindly provided by Yellow Sea Fisheries Research Institute, Chinese Academy of Fishery Sciences. Aprotinin, cytochrome C, salicylic acid, pyrogallol, 2’-Azinobis-(3-ethylbenzthiazoline-6-sulphonate) (ABTS), potassium persulfate, L-reduced glutathione, ACE, hippuric acid, Hip-His-Leu (HHL), Starch, DPP-Ⅳ and Gly-Pro-para-nitroaniline hydrochloride (Gly-Pro-PNA) were purchased from Sigma (St Louis, MO, USA). α-amylase, α-glucosidase and 4-nitrophenyl-α-D-glucopyranoside (PNPG) were purchased from Shanghai Yuanye Bio-Technology Co., Ltd. (Shanghai, China). Bacitracin and H_2_O_2_ were purchased from Aladdin (Shanghai, China). Tetrapeptide GGYR and tripeptide GGG were synthesized by Qiangyao Co., Ltd. (Shanghai, China). Vc was purchased from Sinopharm Chemical Reagent Co., Ltd. (Shanghai, China). HA was purchased from Shandong Freda Bioeng Co., Ltd. (Jinan, China). DPPH• was purchased from Tokyo Chemical Industry (Tokyo, Japan). Other chemicals were of analytical grade and commercially available.

### 3.2. Production and Activity Assay of Protease A69

The protease A69 was produced using recombinant *B. subtilis* in a 15-L fermentor as previously reported [55]. The fermentation medium was composed of 10% maltodextrin, 3.5% soybean meal, 1.3% citrate sodium, 5.5% K_2_HPO_4_, 1.8% MgCl_2_, and 1.0% CaCl_2_. The supernatant after centrifugation of the fermentation culture was concentrated to approximately 40,000 U/mL, which was the enzyme solution of protease A69 to be used in Antarctic krill powder hydrolysis. The enzyme solution of protease A69 was stored at 4 °C before use, and exhibited no obvious activity loss in 1-month storage at 4 °C, and retained more than 90% activity in 6-month storage at 4 °C.

The activity of protease A69 was determined by the Folin–Ciocalteu method as previously reported [54]. Briefly, the reaction mixture containing 100 μL enzyme solution and 100 μL of 2% (*w*/*v*) casein was incubated at 60 °C for 10 min, and then the reaction was stopped by adding 200 μL trichloroacetic acid (0.4 M) into the mixture. After centrifugation of the mixture, 100 μL of the supernatant was mixed with 500 μL of sodium carbonate solution (0.4 M) and 100 μL of the Folin–phenol reagent, which was incubated at 40 °C for 20 min. Then, the OD_660_ of the mixture was measured. One unit (1 U) was defined as the amount of enzyme that released 1 μg of tyrosine from casein at 60 °C and pH 7.0 per minute.

### 3.3. Optimization of the Hydrolytic Parameters of Protease A69 Towards Antarctic Krill Powder

Because the optimal temperature and pH for the activity of A69 were previously determined to be 60 °C and 7.0, respectively [54], all the hydrolytic reactions for hydrolysis parameter optimization were performed at 60 °C and pH 7.0. The hydrolysis parameters of protease A69 towards Antarctic krill powder, including hydrolysis time and enzyme/substrate (E/S) ratio, were optimized based on the methods previously reported [54], with some modifications. Briefly, 5 g Antarctic krill powder in 30 mL ddH_2_O was hydrolyzed by protease A69 at 60 °C and pH 7.0 with a constant stir (180 rpm), and the pH of the mixture was maintained at 7.0 with a 10 M NaOH solution for all optimization reaction. The optimal E/S ratio was determined by hydrolyzing the Antarctic krill powder for 6 h with different E/S ratios (500 U/g, 1000 U/g, 2000 U/g, 3000 U/g, 4000 U/g, 5000 U/g, 6000 U/g). The changes in the E/S ratios were achieved by adding different amounts of enzyme solution of protease A69 to a fixed substrate. The optimal hydrolysis time was determined by hydrolyzing the Antarctic krill powder under the determined optimal E/S ratio (5000 U/g) for different times (1–6 h). After hydrolysis, the mixtures were incubated at 100 °C for 15 min to terminate the reaction, and then centrifuged (4 °C, 16,970× *g*, 30 min). The supernatant and precipitate were, respectively, freeze-dried, and weighed. The powder from freeze-dried supernatant was prepared as a 5 mg/mL solution with ddH_2_O, which was subjected to peptide molecular weight distribution analysis by HPLC (Shimadzu, Kyoto, Japan) gel filtration on a TSK gel G2000 SWXL column (7.8 × 300 mm; Tosoh, Tokyo, Japan), as previously reported [54]. The column was eluted with 45% acetonitrile containing 0.1% (*v*/*v*) trifluoroacetic acid at a flow rate of 0.5 mL/min, and peptide signals were monitored at 220 nm. The weight of the freeze-dried precipitate was used to calculate the hydrolysate yield using the formula: Hydrolysate yield (%) = (W_a_ − W_b_)/W_a_ × 100, where W_a_ and W_b_ were the weight of Antarctic krill powder before hydrolysis and that of the freeze-dried precipitate after hydrolysis, respectively.

### 3.4. Preparation of AKPs with Protease A69

According to the determined optimal hydrolytic parameters, 150 g Antarctic krill powder in 1 L ddH_2_O was hydrolyzed with protease A69 (5000 U/g) at 60 °C and pH 7.0 for 6 h with constant stir (180 rpm). During hydrolysis, the pH of the mixture was maintained at 7.0 with 10 M NaOH solution. After hydrolysis, the AKPs powder was prepared from the mixture through activated carbon treatment, centrifugation, filtration and centrifugal spray drying, as previously described [56].

### 3.5. Characterization of the Prepared AKPs

The prepared AKPs were dissolved in ddH_2_O to prepare 10% (*w*/*v*), 20% (*w*/*v*), and 30% (*w*/*v*) AKPs solutions to investigate the water solubility of the AKPs. Peptide molecular weight distribution of the prepared AKPs was analyzed by HPLC with a 5 mg/mL solution using the method mentioned above. The free and total amino acid composition of the prepared AKPs was determined on an automatic amino acid analyzer, HITACHI 835 (HITACHI, Tokyo, Japan), as previously described [54].

### 3.6. Determination of Protein Content

The protein content of the Antarctic krill powder samples before and after A69 hydrolysis was determined using the colorimetric method according to the National Standard of the People’s Republic of China for determination of protein in foods (GB 5009.168-2016) [105], as previously described [56].

### 3.7. Bioactivity Assays of the Prepared AKPs

The in vitro antioxidant activity of the prepared AKPs was evaluated by determining the scavenging rates of ABTS^+^, DPPH·, ·OH and O_2_^−^·. The scavenging rate of ABTS^+^ was determined according to the National Standard of the People’s Republic of China for the Determination of antioxidant activity of peptides (GB/T 39100-2020) [106]. The clearance rates of DPPH·, ·OH and O_2_^−^· were determined according to the previously reported methods [54]. The ACE-inhibitory activity of the prepared AKPs was determined with the previously reported method [55]. The α-amylase-inhibitory activity [107], α-glucosidase-inhibitory activity [108] and the DPP-IV-inhibitory activity [109] of the prepared AKPs were determined according to the methods previously reported.

The antibacterial effects of the prepared AKPs on *E. coli* and *S. aureus* were determined using the Oxford Cup method [110] with some modification. Briefly, 100 μL of the AKPs water solution (40 mg/mL) was added in the cup standing on the plates containing LB solid medium and *E. coli* or *S. aureus* cells. Kanamycin (1 mg/mL, 100 μL) was used as the positive control and sterile water (100 μL) as the negative control. The plates were incubated at 37 °C. The clear inhibition zones were observed, and their diameters were measured.

### 3.8. Identification of Bioactive Peptides from the Prepared AKPs

The prepared AKPs were subjected to mass spectrometry using a Q Exactive™ Hybrid Quadrupole-Orbitrap™ Mass Spectrometer (Thermo Fisher Scientific, Waltham, MA, USA) in Beijing biotech-pack Scientific Co., Ltd. (Beijing, China). The raw MS files were analyzed and searched against protein databases based on the species in the sample using Byonic. The parameters were set as follows: the protein modifications were carbamidomethylation (C) (fixed), oxidation (M) (variable), and acetyl (N-term) (variable), the enzyme specificity was set to non-specific, the maximum missed cleavages were set to three, the precursor ion mass tolerance was set to 20 ppm, and MS/MS tolerance was 0.02 Da. Only peptides identified with high confidence were chosen for downstream protein identification analysis. In the determined peptide sequences of the prepared AKPs, peptide sequences with bioactivity deposited in databases were searched, including peptide sequences with ACE-inhibitory activity deposited in the AHTPDB database (https://webs.iiitd.edu.in/raghava/ahtpdb/, accessed on 23 March 2025), peptide sequences with antioxidant activity deposited in the AODB database (https://aodb.idruglab.cn/, accessed on 17 March 2025), peptide sequences with antimicrobial activity deposited in the APD database (https://aps.unmc.edu, accessed on 13 March 2025), peptide sequences with α-amylase-inhibitory activity, α-glucosidase-inhibitory activity, or DPP-Ⅳ-inhibitory activity deposited in BIOPEP-UWM (http://www.uwm.edu.pl/biochemia/index.php/pl/biopep, accessed on 23 March 2025).

## 4. Conclusions

In this study, Antarctic krill powder derived from krill oil production was hydrolyzed by marine bacterial metalloprotease A69 to prepare AKPs with multi-bioactivities. Through optimization of hydrolysis parameters, we established a process for AKPs preparation. The prepared AKPs exhibited milky white powder with good water solubility, containing high content of small peptides with molecular weights below 1000 Da (>85%), and 20 amino acids, including 9 human essential ones. Biochemical analyses showed that the prepared AKPs displayed multiple bioactivities, including antioxidant activity, ACE-inhibitory activity, antibacterial activity, α-glucosidase-inhibitory activity and DPP-IV-inhibitory activity, and the functioning concentrations of the AKPs were mostly at the mg/mL level, which are likely higher than the effective physiological concentrations. Further studies are still needed to evaluate the bioactivities of the AKPs at the cellular and animal levels. In addition, based on searching known bioactive peptide sequences deposited in databases, 14 antioxidant peptides, 24 ACE-inhibitory peptides, 2 α-glucosidase-inhibitory peptides, and 10 DPP-Ⅳ-inhibitory peptides were identified from the AKPs. However, it is likely that there are bioactive peptides with unknown sequences in the AKPs, which await further purification, identification and functional evaluation. This study represents the first report on the preparation of AKPs with more than two bioactivities and diverse bioactive peptides. The results indicate that marine bacterial metalloprotease A69 has promising potential in preparing AKPs with good nutritional value and multi-bioactivities, which may have application prospects in functional food, medicine and other industries. This study also offers a possible method for the high value-added utilization of Antarctic krill powder.

## Figures and Tables

**Table 1:** Molecular weight distribution of the AKPs prepared from Antarctic krill powder hydrolysis by A69 at 60 °C and pH 7.0 for 6 h under different E/S ratios.

MW Range (Da)	Content (%)						
	500 U/g	1000 U/g	2000 U/g	3000 U/g	4000 U/g	5000 U/g	6000 U/g
>10,000	0.17	0.12	0.09	0.01	0.01	0	0
5000–10,000	1.78	1.25	0.89	0.74	0.62	0.5	0.49
3000–5000	3.92	3.16	2.57	2.36	2.17	1.92	1.92
1000–3000	24.91	22.97	20.15	18.95	18.43	18.81	17.07
500–1000	28.87	28.95	29.86	29.53	29.42	27.5	29.14
<1000	69.22	72.5	76.31	77.94	78.77	78.77	80.52
<500	40.35	43.56	46.44	48.41	49.35	51.27	51.38

**Table 2:** Molecular weight distribution of the AKPs prepared from Antarctic krill powder hydrolysis by A69 at the E/S ratio of 5000 U/g, 60 °C, and pH 7.0 for different hydrolysis time durations.

MW Range (Da)	Content (%)					
	1 h	2 h	3 h	4 h	5 h	6 h
>10,000	0.02	0.01	0.01	0.01	0	0.01
5000–10,000	0.63	0.49	0.5	0.37	0.11	0.27
3000–5000	2.11	1.8	1.77	1.46	1.1	1.13
1000–3000	21.42	19.42	19.1	13.86	16.95	11.79
500–1000	26.56	31.7	31.28	35.08	29.08	35.14
<1000	75.82	78.28	78.62	84.3	81.84	86.8
<500	49.26	46.58	47.34	49.22	52.76	51.66

**Table 3:** Proportions of peptides with different molecular weights in the prepared AKPs based on HPLC analysis.

MW Range (Da)	Content (%)
>10,000	0
5000–10,000	0.07
3000–5000	0.7
1000–3000	10.86
500–1000	36
<1000	88.37
<500	52.37

**Table 4:** The amino acid composition and content of the AKPs prepared using protease A69 *^a^*.

Amino Acids	Free Amino Acids (%)	Total Amino Acids (%)
Asp *^b^*	0.072 ± 0.001	6.582 ± 0.542
**Thr**	**0.027 ± 0.001**	**2.507 ± 0.205**
Ser	0.034 ± 0.001	2.271 ± 0.123
Glu *^b^*	0.205 ± 0.001	9.802 ± 0.718
Gly	0.196 ± 0.001	2.731 ± 0.225
Ala	0.142 ± 0.001	3.625 ± 0.296
Cys	0.103 ± 0.001	1.723 ± 0.097
**Val**	**0.180 ± 0.001**	**3.157 ± 0.227**
**Met**	**0.115 ± 0.001**	**0.951 ± 0.117**
**Ile**	**0.180 ± 0.002**	**2.312 ± 0.223**
**Leu**	**0.525 ± 0.001**	**4.029 ± 0.404**
Tyr	1.551 ± 0.003	1.825 ± 0.149
**Phe**	**0.659 ± 0.002**	**2.940 ± 0.224**
**Lys**	**0.262 ± 0.001**	**5.308 ± 0.490**
**His**	**-**	**1.532 ± 0.300**
Arg	0.545 ± 0.001	3.873 ± 0.310
Pro	0.150 ± 0.001	8.111 ± 0.496
Trp *^c^*	-	-
Total	4.948 ± 0.001	63.281 ± 0.303

*^a^* Human essential amino acids are shown in bold. The data shown in the table are from triplicate experiments (mean ± SD). *^b^* The detected contents of Asp and Glu include those of potential Asn and Gln, which were converted to Asp and Glu, respectively, during acid hydrolysis. *^c^* Trp was not detectable because it was destroyed in the process of acid hydrolysis.

**Table 5:** Inhibition zone diameters of the AKPs and kanamycin against *E. coli* and *S. aureus ^a^*.

Strain	Sample	Inhibition Zone Diameter (mm)			
		12 h	24 h	36 h	48 h
*E. coli*	kanamycin (1 mg/mL)	23.8 ± 2.0	23.8 ± 2.0	23.8 ± 2.0	23.8 ± 2.0
	H_2_O	-	-	-	-
	AKPs (40 mg/mL)	14.8 ± 1.0	19.5 ± 0.4	27.6 ± 0.6	29.9 ± 0.8
*S. aureus*	kanamycin (1 mg/mL)	18.2 ± 3.0	18.2 ± 3.0	18.2 ± 3.0	18.2 ± 3.0
	H_2_O	-	-	-	-
	AKPs (40 mg/mL)	-	-	-	-

*^a^* The data shown in the table are from triplicate experiments (mean ± SD).

**Table 6:** The antioxidant peptides identified from the prepared AKPs.

Sequence	Molecular Weight (Da)	Source	Antioxidant Activity	References
FL	278.35	Milk Protein	-	[60]
HL	268.31	Designed Peptide	-	[61]
LY	294.34	Soybean Protein	-	[62]
FSL	365.42	Egg White Protein	Oxygen radical absorbance capacity-fluorescein value, <0.022 μmol of Trolox equivalent per μmol of peptide.	[63]
TVM	349.44	Skipjack Tuna	TVM presented scavenging activity on DPPH radical (EC_50_ values of 0.537 ± 0.026), hydroxyl radical (EC_50_ values of 0.942 ± 0.0.067), and superoxide anion free radical (EC_50_ values of 1.069 ± 0.063).	[64]
WAF	422.48	Palm Kernel Cake Hydrolysates	DPPH radical dot radical scavenging activity: 71 ± 2.22%, IC_50_ (μM) = 1.360; Metal chelating activity: 41 ± 1.08%, IC_50_ (μM) = 0.002.	[65]
YMY	475.56	Synthesis Peptide	-	[66]
YYG	401.41	Synthesis Peptide	-	[67]
LVPK	455.59	Black Soybean	-	[67]
SGGY	382.37	Walnut	-	[68]
LKYPI	632.79	Casein-Derived Bioactive Peptides	-	[69]
WDDMEK	822.88	Marine Bivalve	The peptide showed scavenging activity on hydroxyl radical with IC_50_ of 182.4 μM.	[70]
IIAPPER	794.93	*Gryllodes sigillatus*	ABTS^+^ scavenging (EC_50_ mg mL^−1^): 15.62 ± 0.1; DPPH• scavenging (EC_50_ mg mL^−1^): 1.01 ± 0.02; Fe^2+^ chelating activity (EC_50_ mg mL^−1^): 0.142 ± 0.05; Reducing power (Abs700): 0.148 ± 0.01ab LOX inhibitory activity (IC_50_ mg mL^−1^): 8.21 ± 0.04; COX inhibitory activity (IC_50_ mg mL^−1^):8.16 ± 2.22.	[71]
NWDDMEK	936.98	*Scomberomorus niphonius*	-	[72]

**Table 7:** The ACE-inhibitory peptides identified from the prepared AKPs.

Sequence	Molecular Weight (Da)	Source	IC_50_ (μmol L^−1^)	References
PT	216.22	Milk hydrolysate	-	[73]
PT	216.24	Chicken (*Gallus gallus*)	-	-
PT	216.24	Bovine (*Bos taurus*) β-caseins	-	-
PT	216.24	Pork sarcoplasmic proteins	-	[74]
PT	216.24	Cereals storage protein	-	[75]
PT	216.24	Bovine lactoferrin (*Bos taurus*)	-	[76]
SL	218.25	-	-	[77]
LL	244.33	-	-	[77]
LL	244.33	-	-	[78]
HL	268.30	-	3200	-
HL	268.32	Pork sarcoplasmic proteins	-	[74]
HL	268.32	Cereals storage protein	-	[75]
HL	268.32	Bovine (*Bos taurus*) β-caseins	-	[76]
HL	268.32	-	-	[79]
HL	268.32	-	-	[77]
HL	268.32	-	-	[80]
IF	278.34	-	930	[81]
FL	278.35	Rapeseed (Canola meal defatted)	1.33	[82]
FL	278.35	Rapeseed (Canola meal defatted)	1.33	[83]
FL	278.35	-	-	[77]
FL	278.35	-	<20,000	[78]
IF	278.35	Chicken (*Gallus gallus*)	-	-
IF	278.35	Bovine β-caseins	-	-
IF	278.35	Pork sarcoplasmic proteins	-	[74]
IF	278.35	Cereals storage protein	-	[75]
IF	278.35	Soybean Sauce	65.8	[84]
IF	278.35	Cereals (Wheat (Gamma-gliadin from wheat))	-	[76]
IF	278.35	-	-	[79]
IF	278.35	Soybean (Salt-free soy sauce)	65.8	[85]
IF	278.35	Royal jelly	1.67–930	[85]
IF	278.35	-	-	[77]
IF	278.35	-	-	[86]
IF	278.35	-	<20,000	[78]
IF	278.35	-	-	[80]
LY	294.00	Fish (Sardine (*Sardina pilchardus* muscle))	38.5	[87]
LY	294.00	Amaranth (*Amaranthus hypochondriacus*)	-	[88]
LY	294.00	Cereals (Maize (*Zea mays*))	-	[89]
LY	294.33	Fish (Sardine muscle)	18	[87]
LY	294.35	Bovine (*Bos taurus*) β-caseins	-	-
LY	294.35	Pork sarcoplasmic proteins	-	[74]
LY	294.35	Cereals storage protein	-	[75]
LY	294.35	Fish (Sardine)	38.5	[90]
LY	294.35	Rapeseed proteins	110	[91]
LY	294.35	Bovine lactoferrin (*Bos taurus*)	-	[76]
LY	294.35	Bovine β-caseins	-	[76]
LY	294.35	Egg proteins	6.8	[92]
LY	294.35	Milk	-	[93]
LY	294.35	-	38.5	[94]
LY	294.35	-	-	[77]
LY	294.35	-	<20,000	[78]
LY	294.35	-	-	[80]
FF	312.37	-	-	[77]
LLP	341.00	Cereals (Maize (*Zea mays*))	57	[95]
LLP	341.00	Amaranth (*Amaranthus hypochondriacus*)	-	[88]
LLP	341.00	Cereals (Maize (*Zea mays*))	-	[89]
LLP	341.44	Alpha-zein	57	[89]
LLP	341.45	Cereals storage protein	-	[75]
LLP	341.45	Cereals (Rye)	57	[96]
LLP	341.45	Cereals (Wheat (α/β-Wheat gliadin))	-	[76]
LLP	341.45	-	57	[94]
KLP	356.47	-	-	[80]
FGF	369.42	-	-	[86]
FGF	369.42	-	-	[78]
FGF	369.42	-	-	[80]
LNF	392.46	Soybean proteins	-	[82]
MPF	393.50	Egg	6.59–27.38	[85]
MPF	393.50	Egg (cooked egg protein)	17.98	[86]
GVGY	394.43	Silkworm fibroin	35	[97]
GVGY	394.43	Silkworm fibroin	-	[86]
GVGY	394.43	-	-	[80]
YYG	401.42	-	-	[86]
YYG	401.42	-	-	[78]
YYG	401.42	-	-	[80]
VRF	420.51	Soybean proteins	1.3	[82]
LSLP	429.00	-	-	[89]
VFPS	448.51	Cereals (Finnish)	0.46	[98]
VFPS	448.52	Synthesized	0.46	[99]
FLPP	473.00	Cereals (Maize (*Zea mays*))	-	[89]
LIYP	504.61	Human (Human β-casein)	10	[100]
LIYP	504.63	Human (Synthetic peptide of Human β-casein)	10	[100]
LIYP	504.63	Milk	10	[101]
LIYP	504.63	-	10	[94]
LIYP	504.63	-	-	[80]
LIYP	505.00	Milk (human β-casein)	10	[100]
VLPIP	537.69	Human (Human β-casein)	31	[100]
VLPIP	537.70	Human (Synthetic peptide of Human β-casein)	31	[100]
VLPIP	537.70	-	31	[100]
VLPIP	537.70	-	31	[94]
VLPIP	538.00	Milk (human β-casein)	31	[100]
VRYL	549.65	-	24.1	[76]
VRYL	549.67	Milk-Cheese (Sheep milk and cheeses proteins)	24.1	[102]
VRYL	549.67	Cheese (Manchego)	24.1	[83]
VRYL	549.67	Cheese (Manchego)	24.1	[103]
VRYL	550.00	Cheese (Manchego cheese)	-	[104]
IYEGY	643.69	Meat protein	<10	[82]

**Table 8:** The α-glucosidase-inhibitory identified from the prepared AKPs.

Sequence	Molecular Mass (Da)	IC_50_ (µmol L^−1^)
LDNFR	663.72	9.21
IIAPPER	794.94	28.75

**Table 9:** The DPP-Ⅳ-inhibitory peptides identified from the prepared AKPs.

Sequence	Molecular Mass (Da)	IC_50_ (µmol L^−1^)
PT	216.23	-
SL	218.25	2517.08
HA	226.23	-
LL	244.33	-
PM	246.33	-
HL	268.31	143.19
FL	278.35	399.58
IIAP	412.52	-
VPIP	424.53	54.69
FDPF	524.56	-

## Data Availability

The original data presented in the study are included in the article; further inquiries can be directed to the corresponding author.
